# The role of repetitive transcranial magnetic stimulation in reduction of opioid craving: a single-blinded randomized sham-controlled clinical trial

**DOI:** 10.1186/s43045-022-00265-2

**Published:** 2022-12-28

**Authors:** Hanan H. Elrassas, Mahmoud H. Morsy, Yasser M. Abdelrazek, Amany H. El Rasheed, Noha A. Saad, Lobna A. Azzam

**Affiliations:** Okasha Institute of Psychiatry, Department of Psychiatry, Faculty of Medicine, Ain Shams University, 10 Hamed Street, El Abassia, Ramses Street extension, P.O. box 11657, Dair Al-Malak, Cairo, Egypt

**Keywords:** Opioids, Heroin, Tramadol, Craving, TMS, Treatment, Dorsolateral prefrontal cortex

## Abstract

**Background:**

Opioid use disorder (OUD) poses a great concern due to problems associated with their abuse as well as fatal and non-fatal overdose consequences. Craving has a complex relationship with opioid use and relapse. Developing new, effective treatments for substance use disorders (SUDs), including opioid use disorders is crucial. This study aimed to assess the effect of 18 sessions of high-frequency (HF) repetitive transcranial magnetic stimulation (rTMS) over the left dorsolateral prefrontal cortex (DLPFC) on opioid craving in 26 OUD male patients compared to sham rTMS in 26 OUD patients, during early abstinence, with craving assessment using brief substance craving scale (BSCS) for tramadol and morphine and heroin craving questionnaire (HCQ) for heroin craving.

**Results:**

There was a statistically significant reduction in craving scores in the group of OUD patients receiving real rTMS compared to those receiving sham rTMS for both BSCS (*p* value = 0.044) and HCQ (*p* value=0.002). A statistically significant positive correlation was revealed between the number of hospital admissions and the mean scores of post-rTMS HCQ scores (*r*=0.05, *p* value= 0.040).

**Conclusions:**

A high frequency of 10-Hz rTMS over the left DLPFC for 18 treatment sessions reduced craving OUD patients during early abstinence. These preliminary results suggest that 10-Hz rTMS of the left DLPFC may be used in the treatment of OUD, so our study recommends that the use of rTMS in the reduction of opioid craving in early abstinence.

**Trial registration:**

Unique identification number PACTR202206487514449 in the Pan African Clinical Trial Registry retrospectively (www.pactr.org) registered on 10 June 2022.

## Background

Substance use disorder (SUD) is one of the most prevalent neuropsychiatric disorders. Over the past year, around 275 million people have used drugs; up by 22% from 2010. By 2030, demographic factors project the number of people using drugs to rise by 11% around the world and as much as 40% in Africa alone. Among the estimated 275 million past-year users of any drug, approximately 36.3 million almost (13%) are estimated to suffer from drug use disorders, meaning that their drug use is harmful to the point where they may experience drug dependence and/or require treatment. This corresponds to a prevalence of drug use disorders of 0.7% globally among the population aged 15–64 [1]. Craving is a core feature of substance use disorders, as evidenced by its recent addition to the diagnostic criteria for these disorders in the fifth edition of the Diagnostic and Statistical Manual of Mental Disorders (DSM-V) (DSM-5). It entails an irresistible urge to use or intense thoughts about wanting the drug [2, 3]. TMS is a noninvasive neuromodulatory technique increasingly used in clinical and research practices around the world with rising popularity over the past 2 decades. Although originally developed as a diagnostic tool, TMS can transiently or lastingly modulate cortical excitability (either increasing or decreasing it) via the application of localized magnetic field pulses. This and other neurobiological effects can be leveraged for therapeutic applications in neurology, psychiatry, and rehabilitation. rTMS is a tool that can potentially induce dopamine (DA) release and long-lasting changes in neural excitability. High-frequency rTMS at the left DLPFC has been shown to modulate cue-induced cravings, hypothetically due to mimicking actions similar to substances of abuse on the brain reward system [4–6]. The dynamic plasticity of the meso-cortico-limbic pathway is central to addiction, particularly the maladaptive changes that occur within the glutamatergic and dopaminergic systems, and offers a compelling target for therapeutic interventions that can modulate circuit activity. rTMS allows for the non-invasive modulation of these circuits [7]. Opioid cessation involves repeatedly choosing between the immediate reward of using and other options in the context of fluctuating neurobiological, environmental, and cognitive influences. Relapse might reflect a situation where the DLPFC is insufficiently activated to exert effective control on the urge to use Opiates. Modulation of the cellular mechanisms and circuitry involved in this process may be a treatment target for the development of a new aid to cessation treatment [8].

This study proposes that combining a course of 10 Hz rTMS of the left DLPFC decreases craving severity in opioid substance users. However, little is known about its feasibility and potential efficacy. This study is carried out in real-life conditions to assess the role of using rTMS for craving reduction on a scalable level. This study was necessary to try and achieve a refined, effective, and scalable transcranial magnetic stimulation treatment protocol that could prove to be a valuable aid to cessation treatment modalities with fewer side effects and possibly, a better effect.

## Methods

### Study design and setting

This study was a prospective randomized, single-blind sham-controlled, clinical trial with an allocation ratio of participants 1:1. The study was conducted at the Okasha Institute of Psychiatry, Faculty of Medicine, Ain Shams University, located in Eastern Cairo. The study participants were recruited from the substance use disorder clinic and those admitted to the Institute of Psychiatry, in the addiction ward.

### Study subjects

The study targeted males with a diagnosis of SUD, seeking help mainly for opioids. According to the MedCalc computer program (a computer program for medical statistics), the number of subjects was 26 patients diagnosed with substance use disorder (dependence syndrome) as group 1 (active intervention group) and 26 patients diagnosed with substance use disorder (dependence syndrome) as group 2 (sham intervention group). The effect size was used in this exploratory study to estimate the sample size. Group sample sizes of 26 patients in the active intervention group and 26 in the sham intervention group were to achieve 80.75% power to reject the null hypothesis of zero effect size when the population effect size is relatively large (=0.80) and the significance level (alpha) is 0.050 using a two-sided two-sample equal-variance *t* test. The main methods of recruitment included suggesting rTMS as a potential therapeutic method under trial at the Ain Shams University substance dependence treatment clinic. Patients were then randomly allocated in the study arms using a research randomizer, a computer-based random number generator that uses the “Math random” method within the JavaScript programming language as the core method for generating random numbers. Two sets consisting of 26 participants were generated with a random sequence. The sets were generated and sent to operators conducting the rTMS sessions. Subjects eligible for the trial were randomized according to the generated sequence by the operators.

### Inclusion and exclusion criteria

Male patients were included with an age range of 18–65 years old with a diagnosis of opioid use disorder according to the DSM-5 in early abstinence (early abstinence starting 1 week up to 1 month); all participants were approached during this period of time (early abstinence), with exclusion in case of the presence of other serious mental illness (e.g., psychotic disorders, bipolar affective disorder, depression with psychotic features), any other serious medical illness, previous treatment with repetitive, transcranial magnetic stimulation, and patients with contraindications to rTMS as cardiac patients with a pacemaker. All participants were 100 % abstinent throughout the trial period regarding all substances except tobacco, and patients had variable durations of OUD with a variable number of admissions ranging from a year up to 10 years of dependence; this was not considered as an exclusion or inclusion criteria. During the recruitment, in patients reporting intake of substances other than opioids, such as cannabinoids, only those with symptoms not mounting up to a diagnosis of a cannabinoids use disorder were included, and if symptoms mounted up to reach a diagnosis of another comorbid substance use disorder, the participant was excluded.

### Procedure and data collection tools

The participants were assessed using a clinical psychiatric semi-structured interview of the Okasha Institute of Psychiatry, Ain Shams University, which examined demographic data, psychiatric history, examination, and medical history, and the Arabic version of SCID-I [9, 10] was used to screen for psychiatric illnesses other than OUD. The diagnosis of dependence was according to the criteria of the DSMV 5 classification. Consent was taken from patients using written informed consent then the following tools were used:

#### The heroin craving questionnaire (HCQ)

The HCQ was designed according to five theoretically distinct conceptualizations of drug craving: (1) desire to use, (2) intention to use, (3) anticipation of positive outcome, (4) anticipation of relief from withdrawal or dysphoria, and (5) lack of control over use. There is a 14-item version available that contains a subset of items from the 45-item version, and this was the version used in the study. Self-rated responses to each item are recorded on a seven-point Likert scale. A total craving score can be derived from the sum of the 14 individual items. Each item is scored on a scale ranging from 1 for strongly disagree to 7 for strongly agree. Items 1, 5, 8, 9, 10, and 14 are reverse scored. For the total score, the sum of all 14 questions is calculated and divided by 14, for the higher order factor score, the sum of all items except item 1 is calculated and divided by 13 instead, both scores were taken into consideration during the data collection of the study and referred to as HCQ-14 for the 14-item score and HCQ-13 for the higher-order factor score. The higher-order factor score can be used as a uni-dimensional assessment of craving; it correlates with the total score on the 45-item HCQ and with visual analog scales for crave, want, and need [11, 12]. We translated the HCQ into Arabic with translation and back translation by 2 independent translators.

#### The brief substance craving scale (BSCS)

The BSCS is a 16-item, self-report instrument that assesses craving for cocaine and other substances of abuse over a 24-h period. We used the 8-item version with 4 self-report questions regarding 2 potentially craved drugs, measuring the intensity, frequency, length, and number of times for cravings. Each question has 5 answers scored from 0 to 4, 0 being the least and 4 being the highest. The last question inquires about the number of cravings within the past 24 h. The total score is by summing the totals of all questions [13, 14]. We translated BSCS into Arabic with translation and back translation by 2 independent translators.

#### Beck’s depression inventory

The Beck Depression Inventory (BDI) is a 21-item, self-report rating inventory that measures characteristic attitudes and symptoms of depression (Beck, et al., 1961). The BDI has been developed in different forms, including several computerized forms, a card form, the 13-item short form, and the more recent BDI-II [15]. For subclinical depression, only scores ranging from 0 to 13 are taken into account. The Arabic version of the BDI by Abdel-Khalek will be used [16].

#### Repetitivetrans cranial magnetic stimulation (rTMS)

The rTMS will be administered using a Magventure R 30 stimulator with a 75-mm Fig. 8 coil. A total of 18 sessions will be administered, day after day for a total duration of 6 weeks. Patients will be stimulated at a frequency of 10 Hz in 100 trains with a number of 20 pulses per train with an inter-train interval of 15s. each session would last about 13 min. Stimulation intensity will be done at 90% of the motor threshold. The F3 location from the EEG 10–20 system will be used to target the left dorsolateral pre-frontal cortex [17]. For placebo stimulation, a sham coil will be used.

### Methodological and procedural considerations

Medications prescribed included antidepressants as well as mood stabilizers and opioid antagonists (Naltrexone); not all patients received the same pharmacotherapy, yet there was no statistically significant difference or discrepancy between the cases and the control as regards the patterns of pharmacotherapy, hence the inter-individual variation of medication subtype and compliance was disregarded as a confounding factor.

Patients were receiving standard protocol at the institute which entails tailored pharmacotherapy, individual CBT sessions as well as family education, and there were no group therapies held at the time on account of COVID-19 restrictions.

### Statistical analysis

Descriptive statistics were used to express sociodemographic and clinical characteristics. The appropriate statistical tests such as Student’s *t* test and Mann-Whitney *U* test were used to express the group difference for clinical variables on the scores of different scales (for comparison of two groups), and two-way repeated measure ANOVA was run to determine the effect of active rTMS treatment against sham procedure over time. Pearson correlation was used to measure the linear correlation between data. Participants who completed the treatment course and were lost in the course of follow-ups were included. By the end of the data, the study was processed and analyzed by Statistical Package for Social Sciences (SPSS) software version 22.0.

## Results

In this clinical trial, the characteristics of the studied population are explained in Table 1, and a total of 52 Egyptian male OUD patients were studied with a mean age of 33 with age distribution ranging from 18 to 65 years, 92% Muslims, 67% living in urban areas, and 25% having technical diplomas while 20% were illiterate and 40% reported being single while 23% were divorced.

**Table 1:** Sociodemographic characteristics of the male OUD patients studied population

		***n*** (52)	%
**Age in years**	Mean ± SD	33	
	Range	18 - 65 years	
**Gender**	Male	52	100%
	Female	0	0%
**Religion**	Muslim	48	92%
	Christian	4	8%
**Residence**	Urban	35	67%
	Rural	17	33%
**Education level**	Illiterate	10	20%
	Read & write	9	17%
	Secondary	8	15%
	Technical diploma	13	25%
	University	12	23%
**Marital status**	Single	21	40%
	Married	10	19%
	Divorced	12	23%
	Separated	8	15%
	Widowed	1	2%

*N* number, *%* percentage, *SD* standard deviation

Table 2 illustrates that among the total of 52 participants of the study, 21 had no prior history of admission, with a total of 12 patients reporting being admitted once before, followed by a total of 10 being admitted twice, and only 3 being admitted 4 times along the course of their illness, with no statistically significant difference regarding the frequency of admission between both study arms (*p* value = 0.366).

**Table 2:** Description and comparison of the number of hospital admissions for the participating patients among both study arms

Number of admissions	Sham vs real rTMS							
	Real		Sham		Total			
	***N***	%	***N***	%	***N***	%	***X***^**2**^	***P value***
**No**	13	45.83	10	38.46	21	42.00	4.308	0.366
**One**	3	12.50	9	34.62	12	24.00		
**Two**	5	20.83	5	19.23	10	20.00		
**Three**	3	12.50	1	3.85	4	8.00		
**Four**	2	8.33	1	3.85	3	6.00		
**Total**	26	100.00	26	100.00	50	100.00		

*N* number, *%* percentage, *X*^2^ chi square

Within the study population, 26.9% of the total 52 participants reported symptoms of subclinical depression of varying severity using BDI-II prior to the initiation of TMS intervention, yet there was no statistically significant difference regarding the BDI-II scores between both groups as seen in Table 3.

**Table 3:** Screening for sub-clinical depressive symptoms in the study population and a comparison between patients in both study arms

Subclinical depression	Sham vs real rTMS							
	Real		Sham		Total			
	***N***	%	***N***	%	***N***	%	***X***^**2**^	***P*** value
**No**	19	73.08	19	73.08	38	73.08	0.000	1.000
**Yes**	7	26.92	7	26.92	14	26.92		
**Total**	26	100.00	26	100.00	52	100.00		

*N* number, *%* percentage, *X*^*2*^ chi square

Table 4 shows the pattern of different opiates used among the study population regarding the number of substances used, whether single or multiple substances as well as the type of substance used. Among the total study population, 84.6% were polysubstance users, with the most used substance being heroin, at 76.92%, with no statistically significant inter-group variation between both study arms.

**Table 4:** Description and comparison of the pattern of substance use between both study arms of the studied population

**Poly or single substance**	**Sham vs real rTMS**						**Chi-square**	
	**Real**		**Sham**		**Total**			
	***N***	**%**	***N***	**%**	***N***	**%**	***X***^**2**^	***P*** **value**
**Single**	4	15.38	4	15.38	8	15.38	0.000	1.000
**Poly**	22	84.62	22	84.62	44	84.62		
**Total**	26	100.00	26	100.00	52	100.00		
**Main substance**	**Sham vs real rTMS**							
	**Real**		**Sham**		**Total**			
	***N***	**%**	***N***	**%**	***N***	**%**	***X***^**2**^	***P*** **value**
**Heroin**	19	73.08	21	80.77	40	76.92	1.191	0.551
**Tramadol**	6	23.08	5	19.23	11	21.15		
**Morphine**	1	3.85	0	0.00	1	1.92		
**Total**	26	100.00	26	100.00	52	100.00		

*N* number, *%* percentage, *X*^*2*^ chi square, significant, *SD* standard deviation

Table 5 shows craving assessment using the brief substance craving scale (BSCS) for opiates other than heroin both pre-TMS and post-TMS in both real and sham TMS-receiving patient groups, As regards the real r-TMS study arm, a total of 6 patients did not use heroin, hence their craving was assessed using BSCS, and among these, the mean scores for BSCS were approximately 12.57 prior to TMS, falling down to 3.667, with a mean difference being 9 after completion of the 18 treatment sessions with real TMS, which indicates a statistically significant reduction in the mean scores of the group receiving real rTMS (*p* value of 0.001). On the other hand, the group receiving sham TMS had a total of 4 patients and showed a starting mean score in BSCS of 13.4 dropping to 6.75 with a non-statistically significant reduction in craving scores in the study arm receiving sham TMS as indicated by a *p* value of 0.064, indicating a statistically insignificant reduction in the craving after the sham TMS treatment. As regards the intergroup comparison, the pre-rTMS scores show no statistically significant difference between the craving scores for both study groups (*p* value=0.669), yet the post-rTMS scores show a statistically significant difference between the post-rTMS scores of BSCS of the real-rTMS group (3.667) and that of the post rTMS BSCS in the sham group (6.750), with a *p* value of 0.044, suggesting a statistically significant reduction in the craving in the real-rTMS patient group and not in the sham rTMS patient group.

**Table 5:** Craving assessment using the brief substance craving scale (BSCS) for opiates other than heroin both pre-TMS and post-TMS in both real and sham TMS-receiving patient groups

BSCS		Sham vs real rTMS			
		Real	Sham	***T*** test (t)	***P*** value
**Pre**	**Range**	7–17	9–17	−0.441	0.669
	**Mean±SD**	12.571±3.155	13.400±3.286		
**Post**	**Range**	3–5	4–11	−2.385	0.044*
	**Mean±SD**	3.667±0.816	6.750±3.096		
**Differences**	**Mean ±SD**	9.000±2.966	6.250±4.349		
**Paired test**	***P*** **value**	0.001*	0.064		

*N* number, *%* percentage, *X*^*2*^ chi square, *significant, *SD* standard deviation

Table 6 shows craving assessment using the heroin craving questionnaire (HCQ) for heroin users, pre-TMS and post-TMS in both real and sham TMS-receiving patient groups. Heroin craving was measured using the HCQ prior to initiation of r-TMS intervention in both study arms and after completion of 18 sessions, with a calculation of both scores for HCQ-14 and higher order factor HCQ-13. In the real r-TMS group, the mean score before initiation for HCQ-13 was 4.466 and for the HCQ-14 was 4.471, reaching mean scores of 2.669 and 2.692 consecutively post-rTMS with a *p* value of less than 0.001, indicating a statistically significant reduction in craving scores in the real-rTMS-receiving patient study arm after completion of the treatment sessions. As regards scores for the craving of the sham r-TMS-receiving patient group, starting scores of 4.014 are seen in HCQ-13 and 4.022 in HCQ-14, with a final post-treatment score of 3.921 and 3.917 consecutively, and a *p* value of 0.061 indicating a statistically non-significant reduction in the craving scores in this group after completion of the sham rTMS sessions. As regards the intergroup comparison, Table 6 shows the pre-rTMS scores show no statistically significant difference between the craving scores for both study groups, with a *p* value of 0.277 for HCQ-13 and 0.279 for HCQ-14, yet the post-rTMS scores show a statistically significant difference between the post-rTMS scores of both versions of HCQ of the real-rTMS group and that of the post-rTMS scores of both versions of HCQ in the sham group, with a *p* value of 0.002 in both, suggesting a statistically significant reduction in the craving in the real-rTMS patient group and not in the sham rTMS patient group.

**Table 6:** Craving assessment using the heroin craving questionnaire (HCQ-13) for heroin users, pre-TMS, and post-TMS in both real and sham TMS-receiving patient groups

**HCQ-13**		**Sham vs real rTMS**			
		**Real**	**Sham**	***T*** **test (t)**	***P*** **value**
**Pre**	**Range**	2.14–6.15	2.07–5.92	1.102	0.277
	**Mean ±SD**	4.466±1.347	4.014±1.248		
**Post**	**Range**	1.92–4.61	2.01–5.9	−3.386	0.002*
	**Mean ±SD**	2.669±1.062	3.921±1.188		
**Differences**	**Mean ±SD**	1.716±1.447	0.093±0.237		
**Paired test**	***P*** **value**	<0.001*	0.088		
**HCQ-14**		**Sham vs real rTMS**			
		**Real**	**Sham**	***T*** **test (t)**	***P*** **value**
**Pre**	**Range**	2.14–6.14	2.14–5.93	1.099	0.279
	**Mean ±SD**	4.471±1.335	4.022±1.248		
**Post**	**Range**	1.85–4.64	2.12–5.9	−3.324	0.002*
	**Mean ±SD**	2.692±1.061	3.917±1.181		
**Differences**	**Mean ±SD**	1.700±1.396	0.105±0.243		
**Paired test**	***P*** **value**	<0.001*	0.061		

*N* number, *%* percentage, *X*^2^ chi square, *significant, *s* standard deviation

In this study, correlations were done between the number of admissions and the following clinical variables: the age of the participants, BDI-II scores, BSCS scores, and HCQ scores, showing a statistically significant positive correlation between the number of admissions and the post-rTMS scores of HCQ-14, indicating that the higher the number of admissions, the higher the scores of craving after the 18 treatment sessions using HCQ-14, indicating a more severe craving even after the termination of the 18 treatment sessions, illustrated in Table 7 with a *p* value of 0.040.

**Table 7:** Correlation between mean number of admissions of all participants and the mean scores of craving after the termination of the 18 rTMS treatment sessions using HCQ-14 for the total study population

Correlations		
**Spearman’s rho**	**Number of admissions**	
	***R***	***P*** **value**
**HCQ-14 post**	0.335	0.040*

*Significant, *R* spearmen’s rho

## Discussion

In OUD, relapse rates are higher than those for any other drug addiction, with up to 91% of those in recovery experiencing relapses, at least 59% of those would relapse within the first week of sobriety, and 80% would relapse within a month after discharge [18]. An integral part of SUD and OUD is craving with a complex relationship between craving and relapse [19, 20]. Craving is crucial in OUD diagnosis and treatment as acknowledged by its recent addition to the latest version of the DSM; DSM-V [2] international classification of disease systems (ICD-11) [21].

Regarding craving among our study population, a total of 12 patients reported dependence on opiates other than heroin (tramadol and morphine), and a total of 40 participants reported dependence on heroin. The current study showed no statistically significant difference regarding the BSCS, and HCQ, baseline measures between sham and real-study groups but after 18 sessions of rTMS, and there was a statistically significant drop in the post-rTMS scores of the group of patients receiving real rTMS when compared to their initial pre-rTMS scores in both scales. Meanwhile, there was a non-significant change in the post-rTMS scores of the group receiving sham rTMS in comparison to their baseline scores. Also, there was a statistically significant inter-group difference in the final post-rTMS scores between the real-rTMS-receiving group and the sham rTMS-receiving group, at the end of the treatment sessions, indicating that rTMS of 10 Hz at left DLPFC for 18 sessions has a role in reducing craving for opiates (tramadol and morphine) as well as heroin. These findings are in concordance with preliminary behavioral evidence that suggests that acute applications of rTMS reduced drug craving significantly in nicotine; Li et al. [22], alcohol; Mishra et al. [23], heroin; Shen et al. [24], methamphetamine; Liang et al. [25], and cocaine use disorders; Hanlon et al. [26].

As regards OUD in particular, Shen et al. [24] concluded that left DLPFC high-frequency rTMS could significantly reduce craving for heroin in addicts. Twenty male heroin-addicted subjects (aged from 30 to 54 years old) with a heroin use history ranging from 5 to 25 years were recruited and were randomly assigned into a 10-Hz rTMS group and a sham rTMS group (*n* = 10 in each group). The participants rated their craving 5 min before and 5 min after the rTMS or sham stimulation using a subjective craving scale from 0 to 100 to express their heroin craving, 0 as not at all, and 100 as very likely to use. Patients had starting scores of 60 in the rTMS group and 62 in the sham group, continuous rTMS treatment for 10 min for 4 days decreased cue-induced craving in the rTMS group, with the craving score at 25, in contrast to 55 in the sham group. Also, Kandil et al. [27] aimed to assess the efficacy of rTMS and cranial electrotherapy stimulation (CES) on the craving of heroin use disorder. The study included 80 male patients with heroin dependence, with no significance regarding the BSCS baseline measures between all study groups, but after 10 sessions of TMS or CES, these values were decreased with a significant difference in active TMS and CES groups, which reflected a significant reduction of heroin craving and concluded that rTMS and CES are effective non-invasive treatment modalities for the acute reduction of heroin craving with a significant reduction of heroin craving through 3 months after heroin abstinence.

Our study examined the effects of high-frequency rTMS over the left DLPFC, on drug craving, for a duration of 18 sessions, in early abstinence (first month), in a group of 52 male opioid users, comparing the impact of real to sham rTMS. To the best of our knowledge, this study is one of a few to examine the effect of a short course of rTMS on opiate craving. Significant reductions were detected in craving levels over the course of the treatment sessions (6 weeks) in the real rTMS-receiving group compared with the sham rTMS-receiving study arm.

## Conclusions

The use of 10-Hz HF rTMS for 18 treatment sessions day after day over the left DLPFC reduces the craving for opioids in male OUD patients in early abstinence. Transcranial magnetic stimulation remains a promising yet not widely explored territory when it comes to the management of psychiatric disorders and SUD and shows promising results in the treatment of OUD and reduction in craving in early abstinence.

### Limitations

This study targeted only males, with a small study population of a total of 52 participants, and was only single-blinded which impedes generalizing the study results. Also, the cross-sectional nature of this study does not allow follow-up of the patients; hence, large-scale, longitudinal, and interventional studies are required to generalize the results. Currently, there are multiple protocols for clinical studies using rTMS to treat substance use disorders. Nonetheless, there is a general consensus among researchers regarding the cerebral cortex to be stimulated, but substantial study variations limit translation to clinical interventions. Such variations, including laterality of stimulation, rTMS frequency, number of pulses, and number of sessions have made it difficult to attain systematic progress and standardized clinical interventions in SUDs. Cortical target selection, subcortical circuit engagement, optimizing rTMS sequences, rTMS as an adjuvant to existing interventions, manipulating brain state, and selection of outcome measure.

The sample was also small which hindered the validation of the translated tools that were used to assess the severity of craving (HCQ, BSCS).

## Data Availability

The data that support the current study’s findings are available from the corresponding author on request.

## Abbreviations

*X*^2^: Chi-square
%: Percentage
BSCS: Brief substance craving scale
DA: Dopamine
DLPFC: Dorso lateral pre-frontal cortex
DSM-V: Diagnostic and statistical manual 5th edition
HCQ: Heroin craving questionnaire
HF: High frequency
*N*: Number
OUD: Opioid use disorder
rTMS: Repetitive transcranial magnetic stimulation
SCID-I: Structured Clinical Interview for DSM-IV-TR Axis I Disorders
SPSS: Statistical Package for Social Sciences
SD: Standard deviation
SUD: Substance use disorder
TMS: Transcranial magnetic stimulation
